# P123: Hand hygiene in health care environment – from a local action in El Alia to a national programme in Tunisia

**DOI:** 10.1186/2047-2994-2-S1-P123

**Published:** 2013-06-20

**Authors:** MH Dhaouadi, R Hamza, L Essoussi, A Gzara, H Souilah, L Telhig, M Rafrafi

**Affiliations:** 1 El Alia Hospital, El Alia, Tunisia; 2 Regional Directorate of Health Bizerte, Bizerte, Tunisia

## Introduction

In 1996, hand hygiene has been at the heart of the concerns of hospital staff of El Alia. Starting from a local action, the team was heavily involved in the development and implementation of the Tunisian national hand hygiene program that now has more than ten years.

## Objectives

We aim to illustrate the contribution of the team of El Alia to promote hand hygiene at the local, regional and national level and to diffuse an example of a local public health initiative having given birth to a national program.

## Methods

This is a retrospective descriptive study based on consultation documents relating to hand hygiene archived at the hospital in El Alia, at the regional service hygiene Bizerte and the Directorate of Hygiene of the Ministry of Health.

## Results

Three phases could be distinguished:

- Phase I: From 1996 to 2001, marked by the establishment of local action to promote hand hygiene at the hospital in El Alia

- Phase II: From 2002 to 2008, which saw the launch of the national hand hygiene with a strong involvement of the team of El Alia in the development and implementation of this program

- Phase III: From 2009, corresponding to the strengthening of the national hand hygiene again with involvement of the team of El Alia

## Conclusion

This is a beneficial experience on more than one level and rewarding for all. Sustained efforts are needed to achieve sustainable success. A comprehensive assessment should be considered in order to measure the impact of this action.

## Conflict of interest

Not stated

## Disclosure of interest

None declared

